# Comparable Efficacy of Tigecycline versus Colistin Therapy for Multidrug-Resistant and Extensively Drug-Resistant *Acinetobacter baumannii* Pneumonia in Critically Ill Patients

**DOI:** 10.1371/journal.pone.0150642

**Published:** 2016-03-02

**Authors:** Won-Young Kim, Jae-Young Moon, Jin Won Huh, Sang-Ho Choi, Chae-Man Lim, Younsuck Koh, Yong Pil Chong, Sang-Bum Hong

**Affiliations:** 1 Department of Pulmonary and Critical Care Medicine, Asan Medical Center, University of Ulsan College of Medicine, Seoul, Korea; 2 Department of Infectious Diseases, Asan Medical Center, University of Ulsan College of Medicine, Seoul, Korea; Cornell University, UNITED STATES

## Abstract

Tigecycline has *in vitro* activity against multidrug-resistant and extensively drug-resistant *Acinetobacter baumannii* (MDR/XDRAB), and may constitute an alternative therapy for treating pneumonia caused by MDR/XDRAB. The aim of this study was to compare the efficacy of tigecycline-based therapy with colistin-based therapy in patients with MDR/XDRAB pneumonia. Between January 2009 and December 2010, patients in the intensive care unit who were diagnosed with MDR/XDRAB pneumonia and treated with either tigecycline or colistin mono-/combination therapy were reviewed. A total of 70 patients were included in our analysis. Among them, 30 patients received tigecycline-based therapy, and 40 patients received colistin-based therapy. Baseline characteristics were similar in the two groups. Clinical success rate was 47% in the tigecycline group and 48% in the colistin group (*P* = 0.95). There were no differences between the groups with regard to other clinical outcomes, with the exception that nephrotoxicity was observed only in the colistin group (0% vs. 20%; *P* = 0.009). Clinical and microbiological success rates were numerically higher, and mortality rates were numerically lower in combination therapy group than in the monotherapy group. Multivariate analysis indicated that monotherapy was independently associated with increased clinical failure (aOR, 3.96; 95% CI, 1.03–15.26; *P* = 0.046). Our results suggest that tigecycline-based therapy was tolerable and the clinical outcome was comparable to that of colistin-based therapy for patients with MDR/XDRAB pneumonia. In addition, combination therapy may be more useful than monotherapy in treatment of MDR/XDRAB pneumonia.

## Introduction

Nosocomial infections such as pneumonia and bacteremia caused by *Acinetobacter baumannii*, a non-fermentative Gram-negative coccobacillus, are associated with high mortality rates in intensive care units (ICUs) [1, 2]. However, because of the emergence of multidrug-resistant and extensively drug-resistant *A*. *baumannii* (MDR/XDRAB), the management of such infections is difficult [3].

Colistin is currently recommended as a first-line treatment for MDR/XDRAB infections, as MDR/XDRAB remains generally susceptible to colistin [4]. However, the increasing use of colistin monotherapy has been problematic due to the development of resistance [5, 6] and nephrotoxicity [7]. Tigecycline is the first commercially available glycylcyclines, and its use is approved for complicated intra-abdominal and skin/soft tissue infections [8, 9]. Tigecycline has broad spectrum *in vitro* antibacterial activity, including against MDR/XDRAB isolates [10]. In animal models, lung penetration of tigecycline was higher in individuals with *A*. *baumannii* lung infection than in uninfected individuals [11], suggesting a possible role of tigecycline in the treatment of patients with MDR/XDRAB pneumonia. Several non-comparative studies report the use of tigecycline for MDR/XDRAB pneumonia [12, 13]; however, a comparison of tigecycline with colistin for MDR/XDRAB pneumonia is lacking.

In this study, our primary objective was to compare the clinical outcomes of patients with MDR/XDRAB pneumonia treated with a tigecycline-based therapy and those treated with a colistin-based therapy. The secondary objective was to evaluate the efficacy of combination therapy (tigecycline or colistin with other agents for MDR/XDRAB) and compare its efficacy with that of monotherapy.

## Materials and Methods

### Study Design and Patient Selection

This retrospective study was conducted at the Asan Medical Center, a 2,680-bed university-affiliated hospital in Seoul, Korea. We reviewed the medical records of patients admitted to the medical or cardiothoracic ICU between January 2009 and December 2010. Adult patients (≥20 years old) who (i) had a confirmed diagnosis of hospital acquired pneumonia (HAP) or ventilator-associated pneumonia (VAP) caused by MDR/XDRAB and (ii) received either tigecycline or colistin mono-/combination therapy as the initial anti-MDR/XDRAB treatment for at least 3 days were included in analysis. For patients with multiple episodes of MDR/XDRAB pneumonia, only the first episode was included. The exclusion criteria were as follows: concomitant use of tigecycline and colistin; inadequate treatment (<3 days); or combined infection without appropriate antibiotic therapy. The primary study outcome was clinical success rate. Secondary outcomes included: recurrence of infection; microbiological success rate; improvement in Clinical Pulmonary Infection Score (CPIS) and the radiologic score at day 7 compared with baseline (Δ CPIS and Δ radiologic score, respectively); duration of mechanical ventilation (MV), ICU, and hospital stay; nephrotoxicity; and mortality. We also analyzed factors for clinical failure and mortality. The study protocol was approved by the Institutional Review Board (IRB) of the Asan Medical Center (No. 2012–0669). The IRB waived the requirement for informed consent because the study was retrospective, and the patient records were anonymized and de-identified prior to analysis.

### Data Collection and Definitions

Baseline demographic and clinical characteristics were collected and included demographic factors, comorbidities, cause of ICU admission, severity of illness, CPIS and radiologic score, concurrent MDR/XDRAB bacteremia, and treatment prior to receiving tigecycline- or colistin-based therapy. MDR was defined as acquired non-susceptibility to at least one agent in three or more antimicrobial categories, and XDR was defined as non-susceptibility to at least one agent in all but two or fewer antimicrobial categories [14]. Steroid use was defined as corticosteroid therapy being administered within 14 days of infection. HAP was defined as pneumonia that occurs 48 hours or more after admission, which was not incubating at the time of admission. VAP referred to pneumonia arising more than 48 hours after endotracheal intubation. The severity of illness at the time of pneumonia diagnosis was assessed by the Sequential Organ Failure Assessment (SOFA) score [15]. CPIS was used to assess pneumonia severity [16], and radiologic score was also assessed as previously described [17]. Empirical therapy was considered to have been appropriate if at least one effective antimicrobial was included in the initial antibiotic therapy. Combination therapy was defined as at least 3 days of concomitant use of the antimicrobial agents for MDR/XDRAB other than tigecycline or colistin. Clinical success was defined as clinical cure (e.g., resolution of symptoms and signs of infection by the end of therapy) or clinical improvement (e.g., partial resolution of symptoms and signs of infection), and recurrence of infection was defined as occurrence of a new episode of infection at least 72 hours after a preceding episode [18]. Microbiological success was defined as eradication of the pathogen (e.g., no growth of the pathogen in the final culture of specimens during the entire hospitalization) [18]. In patients with normal renal function, nephrotoxicity was defined as a serum creatinine >2 mg/dL, a reduction in the calculated creatinine clearance of 50% compared with the value at the start of therapy, or initiation of renal replacement therapy. In patients with preexisting renal dysfunction, nephrotoxicity was defined as an increase of >50% of the baseline serum creatinine or a reduction in the calculated creatinine clearance of 50% compared with the value at therapy initiation [19].

### Microbiological Studies and Treatment Regimens

The causative MDR/XDRAB pathogen was defined as an isolate from the blood, quantitative culture (≥10^4^ cfu/mL) of a bronchoalveolar lavage (BAL) specimen, semiquantitative culture (moderate or heavy growth) of a bronchoscopic aspirate or BAL specimen, quantitative culture (≥10^5^ cfu/mL) of an endotracheal aspirate, and semiquantitative culture (moderate or heavy growth) of an endotracheal aspirate with white blood cells >25/high power field (HPF) on Gram stain. Bacterial identification was performed using standard methods. Susceptibility testing was done using the Microscan system (Dade Behring, West Sacramento, CA, USA), and results were interpreted according to the Clinical Laboratory Standards Institute (CLSI) guidelines published in 2012 [20]. The minimum inhibitory concentrations (MICs) of tigecycline and colistin were determined using the broth microdilution method, and in this study, isolates with MIC ≤2 mg/L were considered to be susceptible to tigecycline and colistin. The dose of tigecycline was a 100 mg loading, followed by 50 mg every 12 hours. The dose of colistin was 5 mg/kg colistin base activity loading, followed by 150 mg colistin base activity every 12 hours in patients with normal renal function.

### Statistical Analysis

Continuous variables were compared with the Mann-Whitney U test. Categorical variables were compared with the chi-square or Fisher’s exact test. A binary logistic regression was used to identify factors for clinical failure and 30-day mortality. The variables with *P* values <0.10 in the univariate analysis were included in the multivariate analysis by using stepwise backward selection procedures. To prevent multicollinearity, variables with high correlation between each other were controlled. Model discrimination was assessed with the area under the receiver operating characteristic curve (AUC), and model calibration was assessed with Hosmer-Lemeshow test. All tests of significance reported were two-tailed, and a *P* value less than 0.05 was considered significant. Statistical analyses were performed with SPSS version 22.0 for Windows (SPSS Inc., Chicago, IL, USA).

## Results

**Fig 1:**
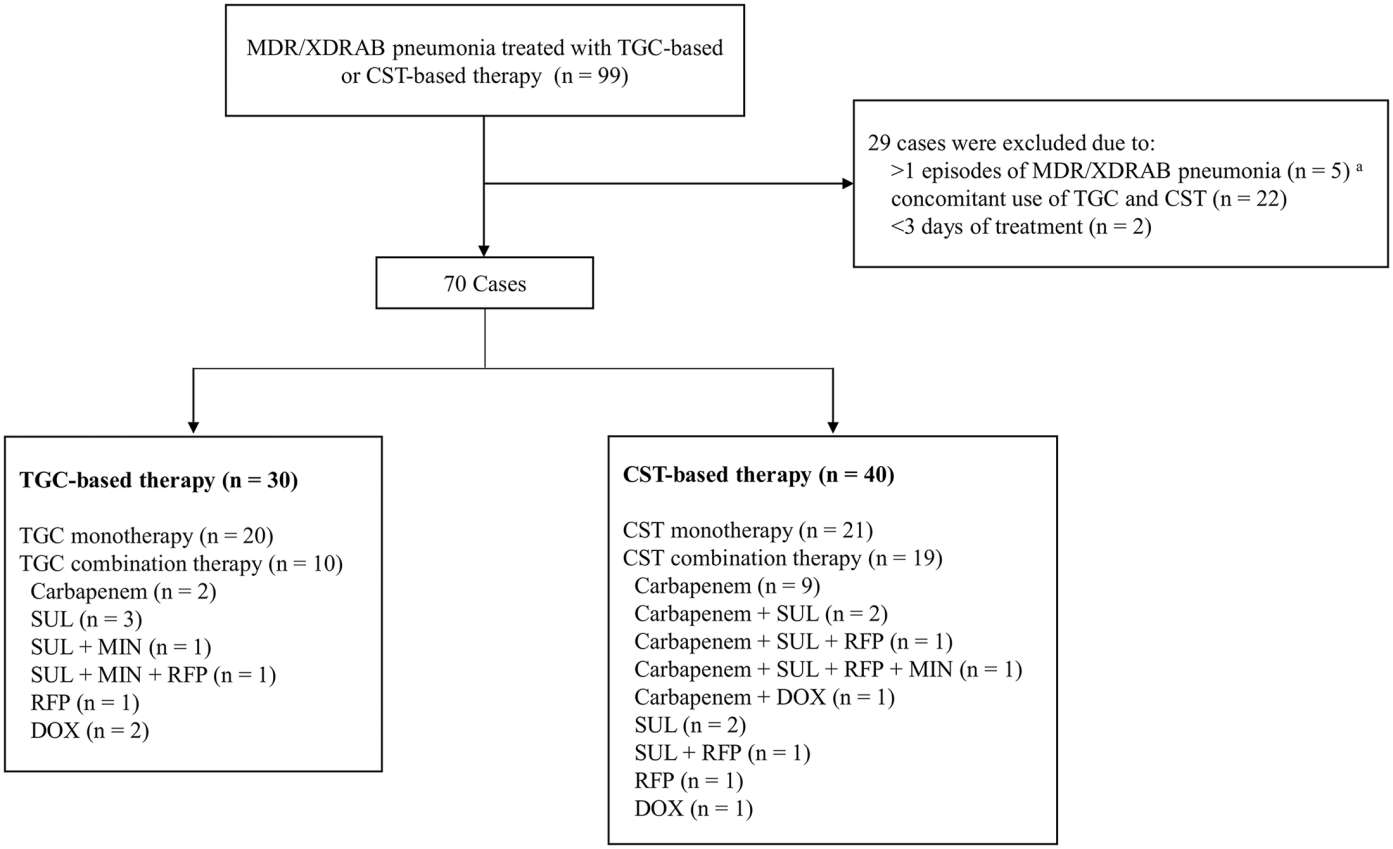
Disposition of MDR/XDRAB pneumonia patients included in the analysis of the impact of tigecycline-based versus colistin-based therapy. MDR/XDRAB, multidrug-resistant and extensively drug-resistant *Acinetobacter baumannii*; TGC, tigecycline; CST, colistin; SUL, sulbactam; MIN, minocycline; RFP, rifampicin; DOX, doxycycline; ^a^ for patients with multiple episodes of MDR/XDRAB pneumonia, the first episode was included in the analysis.

Of the 99 cases of MDR/XDRAB pneumonia during the study period, 29 were excluded for the reasons listed in Fig 1. A total of 30 of the included cases received tigecycline-based therapy, and 40 received colistin-based therapy. Isolates with tigecycline MIC >2 mg/L in the tigecycline group (7%, 2/30) and with colistin MIC >2 mg/L in the colistin group (3%, 1/40) were not excluded in primary analysis. In the tigecycline group, 20 received tigecycline monotherapy and 10 received tigecycline-based combination therapy. In the colistin group, 21 received colistin monotherapy and 19 received colistin-based combination therapy.

### Tigecycline-Based versus Colistin-Based Therapy

Table 1 shows the baseline characteristics of the study patients. Patients in the tigecycline group were older, although this was not statistically significant. There were no significant differences between the two groups with regard to gender, comorbidities, cause of ICU admission, and severity of pneumonia. The concurrent MDR/XDRAB bacteremia was observed in 27% (8/30) in the tigecycline group and 20% (8/40) in the colistin group (*P* = 0.51). Appropriate empirical antibiotic therapy was administered to about half of the patients in each group. The treatment duration was similar between the groups.

**Table 1:** Baseline clinical characteristics of the study patients.^a^

Characteristic	TGC group (n = 30)	CST group (n = 40)	*P*
Age, years	72 (64–76)	67 (57–75)	0.21
Gender, male	24 (80)	30 (75)	0.62
Comorbidity			
Hypertension	13 (43)	12 (30)	0.25
Chronic pulmonary disease	9 (30)	15 (38)	0.51
Diabetes	9 (30)	11 (28)	0.82
Chronic liver disease	2 (7)	5 (13)	0.69
Chronic kidney disease	4 (13)	4 (10)	0.72
Solid cancer	7 (23)	7 (18)	0.55
Hematologic malignancy	3 (10)	6 (15)	0.72
Recent chemotherapy	5 (17)	6 (15)	>0.99
Recent surgery	6 (20)	5 (13)	0.51
Steroid use	19 (63)	21 (53)	0.37
Neutropenia (ANC <1,000 /mm^3^)	1 (3)	3 (8)	0.63
Cause of ICU admission			
Acute respiratory failure	12 (40)	16 (40)	>0.99
Severe sepsis/septic shock	13 (43)	16 (40)	0.78
Postoperative respiratory failure	4 (13)	4 (10)	0.72
VAP	19 (63)	32 (80)	0.12
Mechanical ventilation prior VAP, days	11 (8–17)	16 (9–22)	0.23
At pneumonia diagnosis			
Radiologic score	6.0 (4.0–7.0)	6.0 (4.0–7.0)	0.91
Baseline creatinine, mg/dL	0.8 (0.6–1.4)	0.8 (0.6–1.1)	0.67
Renal replacement therapy	8 (27)	11 (28)	0.94
SOFA score	9.5 (7.0–14.0)	10.0 (8.0–13.5)	0.77
CPIS	6.0 (6.0–7.0)	6.0 (5.5–8.0)	0.88
Concurrent MDR/XDRAB bacteremia	8 (27)	8 (20)	0.51
Appropriate empirical antibiotic therapy	15 (50)	21 (53)	0.84
Treatment duration, days	11 (7–15)	12 (9–19)	0.17

TGC, tigecycline; CST, colistin; ANC, absolute neutrophil count; ICU, intensive care unit; VAP, ventilator-associated pneumonia; SOFA, Sequential Organ Failure Assessment; CPIS, Clinical Pulmonary Infection Score; MDR/XDRAB, multidrug-resistant and extensively drug-resistant *Acinetobacter baumannii*.

^a^ Data are presented as the median (interquartile range) or number (percentage) of patients.

Table 2 shows the clinical outcomes of the study patients. Clinical success rate was 47% (14/30) in the tigecycline group and 48% (19/40) in the colistin group. There were no significant differences between the two groups with regard to recurrence of infection, microbiological success rate, Δ CPIS, or Δ radiologic score. There were also no differences between the groups in duration of MV, ICU, and hospital stay and mortality. Colistin-based therapy was significantly associated with developing nephrotoxicity (*P* = 0.009).

**Table 2:** Clinical outcomes of the study patients.^a^

Outcome/adverse effect	TGC group (n = 30)	CST group (n = 40)	*P*
Clinical success	14 (47)	19 (48)	0.95
Recurrence of infection	2 (7)	4 (10)	0.69
Microbiological success	7 (23)	12 (30)	0.54
Day 7 CPIS	6.0 (4.0–7.0)	5.5 (4.0–7.0)	0.97
Δ CPIS ^b^	-1.0 (-2.0–1.0)	-1.0 (-3.0–1.0)	0.46
Day 7 radiologic score	5.0 (2.0–7.0)	5.0 (3.5–7.5)	0.85
Δ radiologic score ^b^	-1.0 (-2.0–0)	0 (-1.5–1.0)	0.43
MV after pneumonia ^c^, days	10 (5–28)	11 (6–33)	0.71
ICU stay after pneumonia ^c^, days	15 (7–28)	13 (9–39)	0.96
Hospital stay after pneumonia ^c^, days	36 (19–58)	56 (16–111)	0.44
Nephrotoxicity	0	8 (20)	0.009
Mortality			
30-day	10 (33)	12 (30)	0.77
ICU	14 (47)	16 (40)	0.58
In-hospital	15 (50)	20 (50)	>0.99

TGC, tigecycline; CST, colistin; CPIS, Clinical Pulmonary Infection Score; MV, mechanical ventilation; ICU, intensive care unit.

^a^ Data are presented as the median (interquartile range) or number (percentage) of patients.

^b^ Δ was defined as differences between values on day 7 and baseline of pneumonia diagnosis.

^c^ Patients who died within 30 days of the study period were excluded. TGC group (n = 20) vs. CST group (n = 28).

### Monotherapy versus Combination Therapy

The results of additional analysis of the study patients stratified by monotherapy and combination therapy are shown in Table 3. The baseline characteristics did not differ significantly between the groups (see S1 Table for further details). There were no significant differences between the monotherapy and the combination therapy with regard to Δ CPIS, Δ radiologic score, and duration of MV, ICU, and hospital stay. However, there was a trend toward higher clinical and microbiological success rates and lower 30-day, ICU, and in-hospital mortality rates in the combination therapy group.

**Table 3:** Clinical outcomes of monotherapy versus combination therapy.^a^

Outcome	Monotherapy (n = 41)	Combination therapy (n = 29)	*P*
Clinical success	16 (39)	17 (59)	0.11
Recurrence of infection	4 (10)	2 (7)	>0.99
Microbiological success	9 (22)	10 (35)	0.25
Δ CPIS ^b^	-1.0 (-2.0–1.0)	0 (-3.0–1.5)	0.57
Δ radiologic score ^b^	0 (-1.0–0.5)	-1.0 (-2.0–0)	0.42
MV after pneumonia ^c^, days	19 (10–44)	23 (15–50)	0.58
ICU stay after pneumonia ^c^, days	33 (14–47)	28 (17–54)	0.93
Hospital stay after pneumonia ^c^, days	61 (37–125)	84 (49–170)	0.53
Mortality			
30-day	14 (34)	8 (28)	0.56
ICU	21 (51)	9 (31)	0.09
In-hospital	22 (54)	13 (45)	0.47

CPIS, Clinical Pulmonary Infection Score; MV, mechanical ventilation; ICU, intensive care unit.

^a^ Data are presented as the median (interquartile range) or number (percentage) of patients.

^b^ Δ was defined as differences between values on day 7 and baseline of pneumonia diagnosis.

^c^ Patients who died within 30 days of the study period were excluded. Monotherapy (n = 27) vs. Combination therapy (n = 21).

### Risk Factors for Clinical Outcomes of MDR/XDRAB Pneumonia

Table 4 shows the results of univariate and multivariate analysis of risk factors for clinical failure and 30-day mortality. Monotherapy was included in the multivariate analysis because the clinical and microbiological success rates were numerically lower and mortality rates were higher than those of combination therapy in our analysis. Tigecycline use was also included with regard to the recent study findings concerning the use of tigecycline in MDR/XDRAB infections [21, 22]. In the clinical failure model, solid cancer was excluded from further analysis due to multicollinearity with recent chemotherapy. Multivariate analysis that adjusted for variables associated with clinical failure indicated that steroid use, MDR/XDRAB bacteremia, and monotherapy were significantly associated with increased clinical failure rates. MDR/XDRAB bacteremia was a significant prognostic factor for 30-day mortality. On the other hand, tigecycline use was not significantly associated with increased clinical failure or mortality. These models had acceptable discrimination and calibration.

**Table 4:** Univariate and multivariate analysis of factors for clinical failure and 30-day mortality.

Variable	Unadjusted OR (95% CI)	*P*	Adjusted OR ^a^ (95% CI)	*P*
**Clinical failure** ^b^				
Solid cancer ^c^	7.44 (1.52–36.37)	0.01		
Recent chemotherapy	11.85 (1.43–98.59)	0.02	9.53 (0.92–98.55)	0.059
Steroid use	7.25 (2.50–21.05)	<0.001	7.37 (1.95–27.92)	0.003
SOFA score	1.15 (1.02–1.30)	0.02		
Radiologic score	1.27 (1.03–1.56)	0.02	1.31 (1.00–1.70)	0.05
MDR/XDRAB bacteremia	5.42 (1.38–21.22)	0.02	7.18 (1.40–36.85)	0.02
Monotherapy	2.21 (0.84–5.84)	0.11	3.96 (1.03–15.26)	0.046
TGC use	1.03 (0.40–2.67)	0.95		
**30-day mortality** ^d^				
Steroid use	3.70 (1.17–11.63)	0.03	3.91 (0.98–15.62)	0.054
Neutropenia	7.42 (0.73–75.90)	0.09		
SOFA score	1.23 (1.07–1.41)	0.003	1.14 (0.99–1.33)	0.07
MDR/XDRAB bacteremia	8.60 (2.47–29.94)	0.001	6.90 (1.70–27.94)	0.007
Monotherapy	1.36 (0.48–3.85)	0.56		
TGC use	1.17 (0.42–3.23)	0.77		

SOFA, Sequential Organ Failure Assessment; MDR/XDRAB, multidrug-resistant and extensively drug-resistant *Acinetobacter baumannii*; TGC, tigecycline.

^a^ Monotherapy, TGC use, and the other variables with *P* values less than 0.10 (in the univariate analysis) were included in the multivariate analysis.

^b^ Discrimination (AUC = 0.87) and calibration (Hosmer and Lemeshow χ^2^ = 7.37; *P* = 0.39).

^c^ Solid cancer was excluded for the multivariate analysis to prevent multicollinearity.

^d^ Discrimination (AUC = 0.83) and calibration (Hosmer and Lemeshow χ^2^ = 4.93; *P* = 0.77).

## Discussion

The present study indicates that the clinical outcome of tigecycline-based therapy was comparable to that of colistin-based therapy in critically ill patients with MDR/XDRAB pneumonia. A higher rate of nephrotoxicity was observed in the colistin group. Multivariate analysis revealed that monotherapy was associated with increased clinical failure.

Several studies have evaluated the efficacy of tigecycline in MDR/XDRAB infections [12, 13, 23–25]. However, the inclusion of a small number of patients with infections at various sites makes it difficult to establish a role for tigecycline in the treatment of MDR/XDRAB. Chuang et al. recently performed a matched cohort analysis to evaluate the efficacy of tigecycline-based therapy and compare its efficacy with that of colistin-based therapy for the treatment of MDR/XDRAB pneumonia, and demonstrated that the tigecycline-based therapy resulted in higher in-hospital mortality than the colistin-based therapy (61% vs. 44%, respectively). In this study, inferior efficacy of tigecycline might be due to *A*. *baumannii* isolates with higher tigecycline MIC. In the tigecycline group, the proportion of isolates with tigecycline MIC >2 mg/L was 56% (12 of 22 susceptibility-tested cases) and the mortality rate among those isolates was as high as 83% (10/12) [21]. On the contrary, in our study, only two patients in the tigecycline group had pneumonia caused by *A*. *baumannii* isolates with tigecycline MIC >2 mg/L, and the 30-day mortality of the tigecycline group was 33%. Our data indicate that tigecycline-based therapy may be used for MDR/XDRAB pneumonia caused by low tigecycline MIC isolates. In the study by Chuang et al., routine antimicrobial susceptibility testing for tigecycline was not performed (susceptibility testing for only 22 of 84 patients in the tigecycline group of the matched cases). In addition, most of the MDR/XDRAB cases were diagnosed using qualitative sputum culture. *A*. *baumannii* can colonize hospitalized patients and be isolated up to more than 4 months from the respiratory tract [26]. The strengths of our study are: i) the causative pathogen of MDR/XDRAB pneumonia was defined based on quantitative or semiquantitative microbiological data, and ii) routine susceptibility testing for tigecycline was performed for each MDR/XDRAB isolate.

In the present study, the efficacy of tigecycline-based therapy was comparable to that of colistin-based therapy. In previous studies, the efficacy of the approved dose of tigecycline (100 mg loading followed by 50 mg every 12 hours) in pneumonia was questionable, especially in VAP, as antibiotic concentrations in pulmonary epithelial lining fluid relative to those in serum were found to be low [27, 28]. In fact, a recent study revealed that the clinical response seemed to be higher with a higher dose of tigecycline (200 mg loading followed by 100 mg every 12 hours) in pneumonia [29]. In this regard, it would be interesting to compare high dose tigecycline with colistin for the treatment of MDR/XDRAB pneumonia. Combination therapy with more than one agent for MDR/XDRAB could be another option, although several studies reported inconsistent results when combination therapy was used to treat various MDR/XDRAB infections [25, 30, 31]. In our study, clinical failure, microbiological failure, and mortality rates were numerically lower in the combination therapy group than in the monotherapy group. In addition, combination therapy was associated with less clinical failure in multivariate analysis. Despite the relatively small sample sizes of our study, these findings are of great interest, and further large sample size studies should be initiated to define the role of combination therapy in MDR/XDRAB pneumonia.

Tigecycline has pharmacokinetics with a large volume of distribution resulting in a low serum peak concentration [32], and a suboptimal clinical outcome and breakthrough bacteremia during therapy have been observed for tigecycline therapy in MDR/XDRAB bacteremia [33, 34]. In agreement with those observations, our findings showed that the 30-day mortality rate due to concurrent MDR/XDRAB bacteremia was as high as 63% (five of eight patients) in the tigecycline group, although no difference was observed when the mortality rates were compared with those of the colistin group (75%, six of eight patients; *P* > 0.99).

The present study has several limitations. First, the study was single-centered, retrospective, and underpowered. However, our data have important clinical implications because we evaluated the efficacy of tigecycline and colistin, the only two reliably active agents against MDR/XDRAB isolates available at the present time. Second, considerable dropout occurred, due largely to concomitant use of tigecycline and colistin (n = 22). Assessing the efficacy of tigecycline-colistin combination therapy was not one of the objectives of the present study; however, clinical success and 30-day mortality rates of the combination were 64% (14/22) and 18% (4/22), respectively. The results of the assessment were interesting and should be confirmed in future studies. Third, 33% (10/30) of patients in the tigecycline group and 48% (19/40) of patients in the colistin group received combination therapy with more than one agent for MDR/XDRAB. It was thus difficult to attribute clinical and microbiological responses to tigecycline or colistin alone in primary analysis. However, our comparison of effectiveness of monotherapy and combination therapy including multivariate analysis demonstrated that monotherapy may not be effective for the treatment of MDR/XDRAB pneumonia. Further studies are needed to evaluate the efficacy of tigecycline in MDR/XDRAB pneumonia. However, as prospective clinical trials are not programmed in the near future, we believe that, in the meantime, this observational study could aid in the selection of the most appropriate antimicrobial agent for the treatment of pneumonia patients with MDR/XDRAB and, possibly, could also provide a useful background for planning further clinical trials.

In conclusion, our comparison of tigecycline-based therapy with colistin-based therapy for patients with MDR/XDRAB pneumonia indicates that tigecycline was well tolerated and the clinical outcome of tigecycline therapy was comparable to that of colistin therapy. In addition, combination therapy may be more useful than monotherapy in treating MDR/XDRAB pneumonia. Further studies are required to confirm these findings.

## Supporting Information

S1 Table Baseline clnical characteristics of monotherapy versus combination therapy. (PDF)
