# Supplementary material for: Comparable Efficacy of Tigecycline versus Colistin Therapy for Multidrug-Resistant and Extensively Drug-Resistant Acinetobacter baumannii Pneumonia in Critically Ill Patients
Source: PLoS One. 2016 Mar 2;11(3):e0150642. doi: 10.1371/journal.pone.0150642 (PMC4775052; doi:10.1371/journal.pone.0150642)
Supplement: S1 Table — (PDF) [file pone.0150642.s001.pdf]

S1 Table. Baseline clinical characteristics of monotherapy versus combination therapy. [a]

Characteristic	Monotherapy (n = 41)	Combination therapy (n = 29)	*P*
Treatment regimen			0.23
TGC (-based)	20 (49)	10 (35)	
CST (-based)	21 (51)	19 (66)	
Age, years	72 (60–77)	65 (57–71)	0.12
Gender, male	31 (76)	23 (79)	0.72
Comorbidity			
Hypertension	15 (37)	10 (35)	0.86
Chronic pulmonary disease	16 (39)	8 (28)	0.32
Diabetes	11 (27)	9 (31)	0.70
Chronic liver disease	3 (7)	4 (14)	0.44
Chronic kidney disease	4 (10)	4 (14)	0.71
Solid cancer	9 (22)	5 (17)	0.63
Hematologic malignancy	6 (15)	3 (10)	0.73
Recent chemotherapy	7 (17)	4 (14)	>0.99
Recent surgery	9 (22)	2 (7)	0.11
Steroid use	24 (59)	16 (55)	0.78
Neutropenia (ANC <1,000 /mm^3)	3 (7)	1 (3)	0.64
Cause of ICU admission			
Acute respiratory failure	16 (39)	12 (41)	0.84
Severe sepsis/septic shock	15 (37)	14 (48)	0.33
Postoperative respiratory failure	5 (12)	3 (10)	>0.99
VAP	27 (66)	24 (83)	0.12
Mechanical ventilation prior VAP, days	15 (9–21)	13 (9–18)	0.79
At pneumonia diagnosis			
Radiologic score	5.0 (4.0–7.0)	6.0 (4.0–8.0)	0.13
Baseline creatinine, mg/dL	0.7 (0.6–1.2)	0.8 (0.6–1.2)	0.60
Renal replacement therapy	11 (27)	8 (28)	0.94
SOFA score	10.0 (7.0–14.0)	11.0 (7.0–15.0)	0.58
CPIS	6.0 (6.0–8.0)	6.0 (5.0–6.0)	0.06
Concurrent MDR/XDRAB bacteremia	10 (24)	6 (21)	0.72
Appropriate empirical antibiotic therapy	22 (54)	14 (48)	0.66

TGC, tigecycline; CST, colistin; ANC, absolute neutrophil count; ICU, intensive care unit; VAP, ventilator-associated pneumonia; SOFA, Sequential Organ Failure Assessment; CPIS, Clinical Pulmonary Infection Score; MDR/XDRAB, multidrug-resistant and extensively drug-resistant *Acinetobacter baumannii*.

[a] Data are presented as the median (interquartile range) or number (percentage) of patients.
